# The ICOS–ICOSL pathway tunes thymic selection

**DOI:** 10.1111/imcb.12520

**Published:** 2022-01-23

**Authors:** Mengqi Dong, Jinsam Chang, Marie‐Ève Lebel, Noémie Gervais, Marilaine Fournier, Ève Mallet Gauthier, Woong‐Kyung Suh, Heather J Melichar

**Affiliations:** ^1^ Département de Microbiologie, Infectiologie et Immunologie Université de Montréal Montréal QC Canada; ^2^ Immunology‐Oncology Unit Maisonneuve‐Rosemont Hospital Research Center Montréal QC Canada; ^3^ Institut de Recherches Cliniques de Montréal Montréal QC Canada; ^4^ Programme de Biologie Moléculaire Université de Montréal Montréal QC Canada; ^5^ Département de Médecine Université de Montréal Montréal QC Canada

**Keywords:** ICOS, ICOSL, negative selection, thymocytes, thymic antigen‐presenting cells

## Abstract

Negative selection of developing T cells plays a significant role in T‐cell tolerance to self‐antigen. This process relies on thymic antigen‐presenting cells which express both self‐antigens and cosignaling molecules. Inducible T‐cell costimulator (ICOS) belongs to the CD28 family of cosignaling molecules and binds to ICOS ligand (ICOSL). The ICOS signaling pathway plays important roles in shaping the immune response to infections, but its role in central tolerance is less well understood. Here we show that ICOSL is expressed by subsets of thymic dendritic cells and medullary thymic epithelial cells as well as thymic B cells. ICOS expression is upregulated as T cells mature in the thymus and correlates with T‐cell receptor signal strength during thymic selection. We also provide evidence of a role for ICOS signaling in mediating negative selection. Our findings suggest that ICOS may fine‐tune T‐cell receptor signals during thymic selection contributing to the generation of a tolerant T‐cell population.

## Graphical abstract

We show that inducible T‐cell costimulator (ICOS) expression is upregulated as T cells mature in the thymus and that its ligand is expressed by important thymic antigen‐presenting cell populations that regulate negative selection of autoreactive T cells. We provide evidence of a role for the ICOS–ICOS ligand pathway in fine‐tuning T‐cell receptor signals during thymic selection contributing to the generation of a tolerant T‐cell population. Our observations add an additional layer of complexity to the network of cosignaling molecules potentially involved in sculpting the T‐cell receptor repertoire.

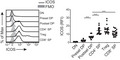

## INTRODUCTION

T cells express an enormous diversity of antigen receptors to recognize the myriad of foreign antigens that they may encounter. Potentially autoreactive T‐cell receptors (TCR) are generated during rearrangement of antigen receptor gene segments during T‐cell development in the thymus. Clonal deletion eliminates many of these autoreactive T cells in the thymus to prevent them from entering the periphery.^1^, ^2^ Broadly, developing T cells scan for self‐antigens presented by major histocompatibility complex (MHC) molecules on thymic antigen‐presenting cells (APC). Thymocytes expressing TCRs with low to moderate affinity to self‐peptide receive survival and differentiation signals (positive selection). Thymocytes bearing a TCR with high affinity for self‐antigens undergo apoptosis (negative selection).^3^ Alternatively, some high‐affinity T‐cell clones can be diverted into immunosuppressive T‐cell lineages that include Foxp3^+^CD4^+^ regulatory T cells (Treg) and provide an additional layer to maintain self‐tolerance.^3^, ^4^

Thymic negative selection occurs at both the CD4^+^CD8^+^ double‐positive (DP) and the more mature CD4^+^ or CD8^+^ single‐positive (SP) stages of T‐cell development.^5^, ^6^ A significant percentage of DP thymocytes expressing TCRs with high affinity to ubiquitous self‐antigens is eliminated in the thymic cortex.^5^, ^7^, ^8^, ^9^ While cortical thymic epithelial cells (cTEC) do not efficiently support clonal deletion, cortical dendritic cells (DC) are sufficient to induce apoptosis of DP thymocytes.^7^, ^10^ Mature CD4^+^ and CD8^+^ SP thymocytes further test their TCRs for reactivity to tissue‐restricted antigens within the thymic medulla. The thymic medulla contains diverse APCs that play distinct roles in T‐cell tolerance.^11^, ^12^ Medullary thymic epithelial cells (mTEC) can express almost all genes.^13^, ^14^, ^15^, ^16^ The tissue‐restricted antigens expressed by mTEC can be cross‐presented by thymic DCs.^17^, ^18^ DCs can also carry antigens acquired in the periphery to the thymus to induce T‐cell tolerance to peripheral antigens.^19^, ^20^, ^21^ In addition, thymic B cells express a unique set of tissue‐restricted antigens to promote negative selection.^22^, ^23^, ^24^

In the thymus, mTEC, thymic DCs and B cells express cosignaling molecules that may modulate thymic selection. Indeed, it has been suggested that TCR signaling alone is insufficient to induce negative selection of all autoreactive thymocytes.^25^ CD28 binds to both CD80 and CD86 and emerged early as a candidate cosignaling molecule necessary for negative selection. While there is substantial evidence that negative selection requires CD28 cosignaling,^26^, ^27^, ^28^ in some conditions, CD28 does not appear to be necessary for clonal deletion.^25^, ^29^, ^30^, ^31^ It is possible that other costimulatory molecules may compensate for the absence of CD28 in these instances and/or complement its function at steady state.^32^, ^33^, ^34^, ^35^, ^36^

Inducible T‐cell costimulator (ICOS) and its unique binding partner, ICOS ligand (ICOSL), belong to the CD28‐B7 superfamily. CD28 and ICOS play complementary, nonredundant roles in effector T‐cell and Treg functions during an immune response.^37^, ^38^ ICOS is not expressed by mature naïve T cells and is only upregulated after activation; ICOSL is expressed on both hematopoietic and nonhematopoietic cells.^37^ In the thymus, ICOS is expressed on some thymocytes and has been implicated in the regulation of innate T‐cell subset development.^39^, ^40^ In addition, ICOSL expression on mTEC may play a role in thymic Treg generation; in an *in vitro* system, ICOSL expression on human mTEC promoted a Treg phenotype via increased interleukin‐2 production by CD4^+^ SP thymocytes.^41^ As interleukin‐2 production by self‐reactive T cells is important for inducing Foxp3 expression on Tregs,^42^ mTEC expressing ICOSL may indirectly lead to the expansion of thymic Tregs. However, reports of ICOSL expression on thymic APCs are inconsistent,^40^, ^43^, ^44^ and it is unclear whether ICOS is implicated in the selection of conventional T cells.

In this study, we re‐examined ICOSL expression on TEC, thymic DCs and B cells, as well as ICOS expression on T‐cell developmental intermediates. ICOSL is expressed by thymic APCs that coexpress other cosignaling molecules and are known to support negative selection. We observed an upregulation of ICOS on DP thymocytes that have received thymic selection signals. Higher levels of ICOS expression are detected on SP thymocytes and Tregs relative to their less mature counterparts, but ICOS is down‐regulated prior to thymic egress. The upregulation of ICOS correlates with the strength of TCR signaling received during T‐cell development. Using ICOSL‐deficient thymic slices, we identify a potential role for the ICOS pathway in fine‐tuning negative selection of developing T cells bearing TCRs with high affinity for self.

## RESULTS

### Inducible T‐cell costimulator ligand is expressed by subsets of thymic antigen‐presenting cells

**Figure 1:**
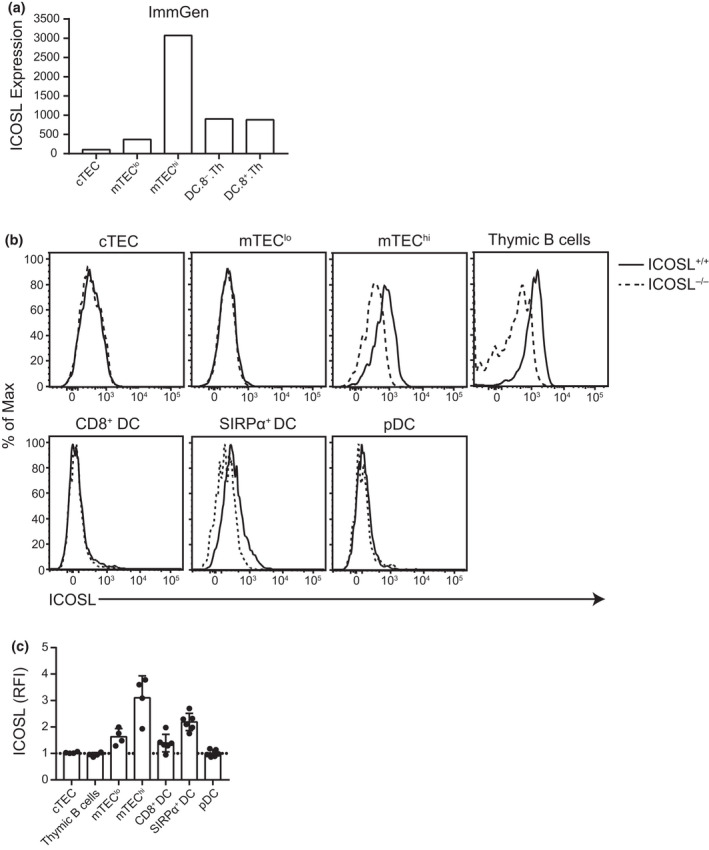
Inducible T‐cell costimulator ligand (ICOSL) is expressed on subsets of medullary thymic epithelial cells (mTEC) and thymic dendritic cells (DCs) as well as thymic B cells. **(a)** Relative levels of ICOSL expression extracted from the ImmGen database on the Ep.8wk.CEChi. Th.v2 [cortical thymic epithelial cells (cTECs)], Ep.8wk.MEClo. Th.v2 [mTEC low (mTEC^lo^)] and Ep.8wk.MEChi. Th.v2 [mTEC high (mTEC^hi^)] as well as DC.8^−^. Th and DC.8^+^. Th (thymic CD8^−^ and CD8^+^ DC) subsets.^47^ **(b)** Representative histograms and **(c)** relative expression of ICOSL from cTEC, mTEC^lo^, mTEC^hi^, thymic B cells, CD8^+^ DC, SIRPα^+^ DC and plasmacytoid DC (pDC) analyzed by flow cytometry. The gating strategies are depicted in Supplementary figure 1. The relative fluorescence intensity for ICOSL expression is normalized to the median fluorescence intensity of each subset from ICOSL^−/−^ mice. Dots indicate individual mice; *n* = 4 or 6 mice from a minimum of two independent experiments.

CD80 and CD86, ligands for CD28, are expressed at high levels on a subset of mTEC that also express high levels of MHC class II (MHC‐II). Thymic DCs and B cells also generally have high expression of CD80, while CD86 expression is lower.^19^, ^22^, ^23^ However, ICOSL expression on the surface of these cells has either not been examined, or the reported results are inconsistent.^40^, ^43^, ^44^ The inconsistencies in the literature may be a result of stromal cell isolation methods that could cleave ICOSL from the cell surface and therefore limit its assessment by flow cytometry.^45^ From microarray data deposited on the Immunological Genome Project Consortium and from published single‐cell RNA‐sequencing data,^46^, ^47^ there is evidence that ICOSL is significantly expressed on CD80^hi^MHC‐II^hi^ mTEC (mTEC^hi^) and thymic DCs (Figure 1a). There is no transcriptomic data available for thymic B cells in this database for direct comparison. To understand which thymic APCs express ICOSL on the cell surface, we tested different thymic stromal cell isolation approaches (Figure 1b, c, Supplementary figure 1a, b). The use of Liberase and DNase I allowed us to detect ICOSL expression and compare its levels on cTEC, CD80^lo^MHC‐II^lo^ mTEC (mTEC^lo^), mTEC^hi^ and thymic CD11c^+^ DC subsets (Figure 1b, c, Supplementary figure 1a, b). ICOSL expression on ICOSL^+/+^ cTEC and mTEC^lo^ are indistinguishable from those of ICOSL^−/−^ origin, suggesting that it is not expressed at significant levels on these populations; by contrast, ICOSL is highly expressed on ICOSL^+/+^ mTEC^hi^ (Figure 1b, c). Among thymic DC subsets, expression of ICOSL is highest on SIRPα^+^ DCs and, on average, lowest on plasmacytoid DCs (Figure 1b, c, Supplementary figure 1a). Similar to their peripheral B‐cell counterparts, we found that thymic B cells also express high levels of ICOSL (Figure 1b, c and Supplementary figure 1c).^48^ Therefore, ICOSL is preferentially expressed on many of the thymic APC populations that also express high levels of other cosignaling molecules such as CD80/CD86 and support negative selection.

### Inducible T‐cell costimulator expression is developmentally regulated in the thymus

**Figure 2:**
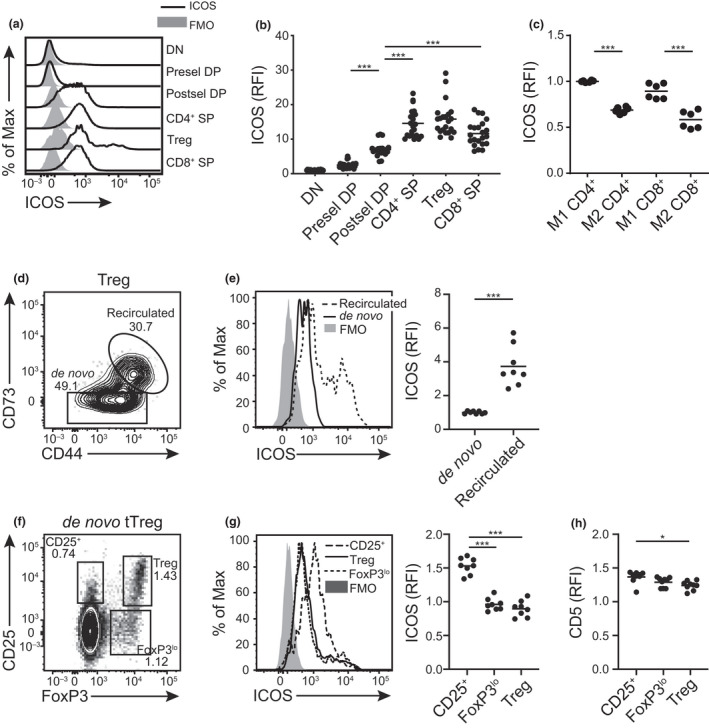
Differential expression of inducible T‐cell costimulator (ICOS) on thymocyte subsets. **(a)** Representative histograms and **(b)** relative expression of ICOS on thymic developmental subsets in wild‐type (WT) adult mice analyzed by flow cytometry: CD4^−^CD8^−^TCRβ^−^ double‐negative (DN), CD4^+^CD8^+^CD69^−^ preselection double‐positive (Presel DP), CD4^+^CD8^+^CD69^+^ postselection DP (Postsel DP), TCRβ^hi^CD25^−^Foxp3^−^CD4^+^ single‐positive (SP), TCRβ^hi^CD4^+^CD25^+^Foxp3^+^ regulatory T (Treg), TCRβ^hi^ CD8^+^ SP. Dots in **b** indicate individual mice (*n* = 22) from a minimum of three independent experiments. Relative fluorescence intensity (RFI) is calculated after normalization to ICOS median fluorescence intensity (MFI) of the DN subset in each experiment. **(c)** Relative expression of ICOS on distinct maturation stages of CD4^+^ SP and CD8^+^ SP thymocytes (CD69^+^MHC‐I^+^ M1 and the more mature CD69^−^MHC‐I^+^ M2). Dots in **c** indicate individual mice (*n* = 6) from two independent experiments. The RFI for ICOS is normalized to the MFI of the M1 CD4^+^ SP subset in each experiment. **(d)** Representative flow plots of recirculated (CD73^+^CD44^+^) and *de novo* (CD73^lo^) thymic Tregs. **(e)** Representative histograms and quantification of ICOS expression on recirculated and *de novo* thymic Tregs. The RFI is normalized to the MFI of *de novo* thymic Tregs in each experiment. **(f)** Representative flow plots for two thymic Treg precursors (CD25^+^ and Foxp3^lo^) gated on *de novo*, nonrecirculated thymocytes. **(g)** Representative histograms and quantification of ICOS expression on thymic Treg precursors and Foxp3^+^CD25^+^ Tregs. **(h)** Quantification of CD5 expression on thymic Treg precursors and mature Foxp3^+^CD25^+^ Tregs. **(g)** and **(h)** The RFI for ICOS or CD5 is normalized to the MFI of the CD4^+^ SP subset in each experiment. Dots in **e**, **g** and **h** indicate individual mice (*n* = 8) from three independent experiments. *P < 0.05, ***P < 0.001, Welch’s *t*‐test **(e)** or Brown–Forsythe and Welch ANOVA followed by Dunnett’s T3 multiple comparisons test **(b, c, g, h)**. FMO, fluorescence minus one control; MHC, major histocompatibility complex; TCR, T‐cell receptor.

It has been long thought that only activated effector T cells and Treg express ICOS. Although recent evidence suggests that ICOS is expressed by some thymocytes, it is still unclear which developing thymocyte populations express this cosignaling molecule.^39^ We examined ICOS expression on TCRβ^−^CD4^−^CD8^−^ double‐negative (DN), CD4^+^CD8^+^CD69^−^ preselection and CD4^+^CD8^+^CD69^+^ postselection DP, mature (TCRβ^hi^) CD4^+^CD25^−^Foxp3^−^ and CD8^+^ SP thymocytes as well as CD4^+^CD25^+^Foxp3^+^ Treg from wild‐type (WT) mice (Supplementary figure 2a); of note, the DN population using this gating strategy will contain a small percentage of γδ T cells. The expression of ICOS is low on DN and preselection DP cells. ICOS expression is upregulated as thymocytes receive positive selection signals in the postselection DP stage, and it is further elevated on CD4^+^ and CD8^+^ SP thymocytes (Figure 2a, b). This suggests the expression of ICOS is upregulated coincident with TCR signals received during T‐cell development. Interestingly, however, ICOS expression begins to decrease from the CD69^+^MHC‐I^+^ (M1) to the more mature CD69^–^MHC‐I^+^ (M2) CD4^+^ and CD8^+^ SP stages of development as TCR sensitivity for self‐peptide diminishes just prior to thymic egress (Figure 2c, Supplementary figure 2a).^49^

In contrast to the homogenous expression of ICOS in postselection DP and SP thymocyte populations, there appears to be a second, small population of Treg with significantly higher expression of ICOS (Figure 2a). To investigate which Treg subpopulations contribute to the heterogeneity in ICOS expression, we first examined ICOS levels on *de novo* and mature Treg that have recirculated to the thymus from the periphery. In our experiments, we used mice between 6 and 12 weeks of age, where often more than 30% of the Treg population within the thymus is composed of mature Treg that have recirculated from the periphery (Figure 2d).^50^ Recirculated Tregs, in general, exhibit an activated phenotype.^50^ Indeed, we observed that the relative fluorescence intensity of ICOS is about fivefold higher on recirculated Tregs than *de novo* thymic Tregs (Figure 2e). In addition, *de novo* Foxp3^+^CD25^+^ thymic Tregs can be derived from either CD25^+^Foxp3^−^ or CD25^−^Foxp3^lo^ CD4^+^ precursors.^51^ After excluding recirculated CD4^+^ T cells (Supplementary figure 2b), we compared ICOS levels between the two thymic Treg precursors with Foxp3^+^CD25^+^ Treg and conventional CD4^+^ SP thymocytes (Figure 2f). Interestingly, CD25^+^ Treg precursors expressed higher ICOS levels, while Foxp3^lo^ Treg precursors and the Foxp3^+^CD25^+^ Tregs expressed lower levels of ICOS (Figure 2g). CD25^+^ Treg precursors are thought to develop from CD4^+^ T cells bearing antigen receptors with a relatively higher affinity for self‐antigen than Foxp3^lo^ Treg precursors.^51^ Indeed, we observed that cell surface expression of CD5, a marker that correlates with the strength of TCR signals,^52^ is higher on CD25^+^ Treg precursors than those that are Foxp3^lo^ Treg progenitors (Figure 2h). These findings indicate that ICOS expression is upregulated during *de novo* thymic Treg development and that it is preferentially expressed on Treg progenitors with higher affinity for self‐antigens.

### Inducible T‐cell costimulator expression correlates with the strength of TCR signals

**Figure 3:**
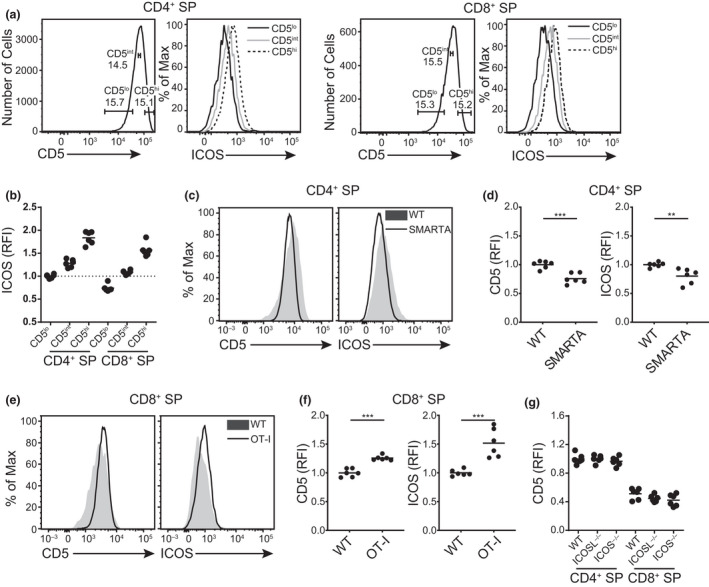
Inducible T‐cell costimulator (ICOS) expression correlates with the strength of T‐cell receptor (TCR) signaling. **(a)** Representative histograms and **(b)** relative ICOS expression on CD4^+^ or CD8^+^ single‐positive (SP) thymocytes gated on the top (CD5^hi^), intermediate (CD5^int^) and bottom (CD5^lo^) 15% of CD5 expression. The relative fluorescence intensity (RFI) of ICOS is nbormalized to the median fluorescence intensity (MFI) of ICOS on CD5^lo^ CD4^+^ SP thymocytes in each experiment. **(c)** Representative histograms and **(d)** relative expression of CD5 (left) and ICOS (right) on CD4^+^ SP thymocytes from nontransgenic wild‐type (WT) mice as compared with those from MHC‐II‐restricted TCR transgenic SMARTA mice. **(e)** Representative histograms and **(f)** relative expression of CD5 (left) and ICOS (right) on CD8^+^ SP thymocytes from WT mice as compared with those from MHC‐I‐restricted TCR transgenic OT‐I mice. **(g)** Quantification of CD5 expression on CD4^+^ SP and CD8^+^ SP thymocytes from WT, ICOS ligand (ICOSL)‐ and ICOS‐deficient mice. The RFI for CD5 is normalized to the MFI of CD4^+^ SP in each experiment. Dots indicate individual mice (*n* = 6) from a minimum of two independent experiments. **P < 0.01, ***P < 0.001, two‐tailed unpaired Student’s *t*‐test.

Among thymic Treg populations, ICOS is preferentially expressed among cells that receive stronger TCR signals during differentiation. In addition, there is some evidence that the level of expression of ICOS on peripheral T cells may be dependent on signal strength.^53^, ^54^ To better understand the correlation of ICOS with TCR signals received during thymic development, we compared the levels of ICOS on CD4^+^ and CD8^+^ SP thymocytes gated on the top and bottom 15% of CD5 expression as well as a CD5 intermediate population (Figure 3a). We observed that CD4^+^ and CD8^+^ SP thymocytes expressing higher levels of CD5 also express higher levels of ICOS (Figure 3a, b and Supplementary figure 3a). We also sought to examine to what extent the positive correlation between CD5 and ICOS holds true in TCR transgenic mouse models where all T cells express a single TCR. We compared CD5 and ICOS levels on mature thymocytes from WT mice with MHC‐II‐restricted SMARTA (Figure 3c, d) and MHC‐I‐restricted OT‐I (Figure 3e, f) TCR transgenic mice. Relative to WT CD4^+^ SP thymocytes, those from SMARTA TCR transgenic mice express lower levels of CD5 (Figure 3c, d, left panels). SMARTA CD4^+^ SP thymocytes also expressed significantly lower ICOS levels than their WT counterparts (Figure 3c, d, right panels). By contrast, CD8^+^ SP thymocytes from OT‐I mice express higher levels of CD5 and ICOS than their counterparts in WT mice (Figure 3e, f). It is also possible that ICOS–ICOSL interactions may, in turn, influence TCR signals in response to self‐antigen at later stages of development. As such, we assessed CD5 levels on thymocytes from WT (ICOS^+/+^ICOSL^+/+^), ICOS^−/−^ and ICOSL^−/−^ mice. Ultimately, we do not detect any statistically significant differences in CD5 levels among conventional CD4^+^ and CD8^+^ SP thymocytes nor Treg from WT *versus* ICOS‐ or ICOSL‐deficient mice (Figure 3g, Supplementary figure 3b). Overall, our results suggest that ICOS expression on thymocytes correlates with the strength of TCR signaling they received during thymic selection but that ICOS signaling during T‐cell differentiation does not globally change the strength of TCR signals perceived during development.

### Inducible T‐cell costimulator may fine‐tune negative selection

We demonstrated that ICOSL is expressed on subsets of APCs predominantly localized to the thymic medulla and corticomedullary region, and that ICOS is upregulated at the postselection DP and SP thymocyte stages. Therefore, we considered the possibility that ICOS–ICOSL interactions may play a previously unappreciated role in the thymic selection outcome of ICOS‐expressing thymocytes. Except for innate T‐cell subsets, it has been suggested that thymic T‐cell development is largely normal in ICOS signaling‐deficient mice^39^, ^40^, ^55^, ^56^; in line with this, we do not observe any consistent abnormalities in the percentages or number of conventional T‐cell and Treg populations in the thymus of ICOS^−/−^ and ICOSL^−/−^ mice as compared with WT (ICOS^+/+^ICOSL^+/+^) controls (Supplementary figure 4a). In addition, there is no difference in the accumulation of thymocyte populations with select markers of strong signals that accompany negative selection (Supplementary figure 4b and data not shown). The one exception is a reduction in a population of CD8^+^ SP thymocytes that express PD‐1 in ICOS‐ and ICOSL‐deficient mice as compared with controls (Supplementary figure 4c).

**Figure 4:**
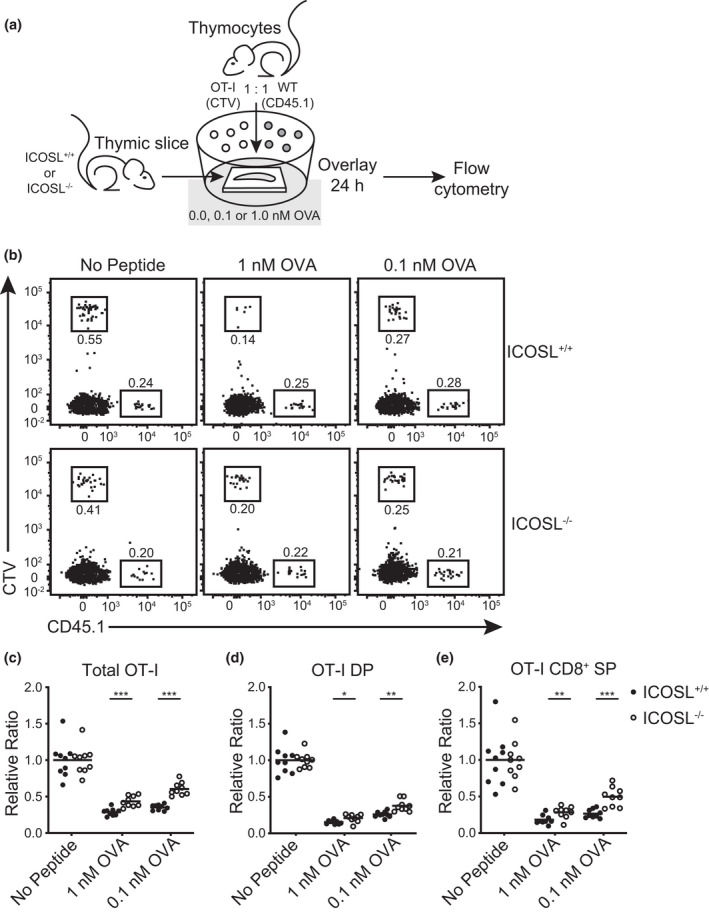
Negative selection of OT‐I T‐cell receptor (TCR) transgenic thymocytes is less efficient in the absence of inducible T‐cell costimulator ligand (ICOSL). **(a)** Schematic representation of experimental setup. CellTrace Violet (CTV)‐labeled total OT‐I thymocytes were mixed at a 1:1 ratio with congenic CD45.1^+^ wild‐type (WT) thymocytes and were overlaid atop thymic slices generated from either ICOSL^+/+^ or ICOSL^−/−^ mice in the presence or absence of OVA peptide (SIINFEKL) at the indicated concentrations. The slices were analyzed by flow cytometry 24 h later. **(b)** Representative flow plots and **(c)** compilation of the relative proportion of total live OT‐I thymocytes normalized to the proportion of congenic CD45.1^+^ thymocytes from each slice; a decrease in the proportion of OT‐I thymocytes indicates negative selection. The relative ratio of **(d)** CD4^+^CD8^+^ double‐positive (DP) OT‐I thymocytes or **(e)** CD8^+^ single‐positive (SP) OT‐I thymocytes. *n* = 9 thymic slices for each condition from three independent experiments, *P < 0.05, **P < 0.01, ***P < 0.001, two‐tailed unpaired Student’s *t*‐test within each peptide concentration condition.

Given that PD‐1 is a marker that has been associated with strong, persistent signals in the thymus and autoreactive TCRs,^27^ we sought to determine whether ICOS–ICOSL interactions influenced negative selection of MHC‐I‐restricted thymocytes. For this, we used TCR transgenic thymocytes that will undergo negative selection in the presence of their cognate antigen in an *in situ* thymic slice system.^57^, ^58^, ^59^, ^60^ We generated thymic slices from ICOSL^+/+^ and ICOSL^−/−^ mice and overlaid the slices with CellTrace Violet‐labeled total OT‐I thymocytes mixed 1:1 with control congenic (CD45.1) WT thymocytes. OVA (SIINFEKL) peptide, the cognate antigen for the OT‐I TCR, was added in the media for the thymic slices (Figure 4a). The thymic APCs can take up the peptides from the media and present them to the overlaid thymocytes. Maximal negative selection is achieved in this model with 1 nM of peptide, and we reasoned that a role for costimulation in negative selection may be more evident in conditions where peptide availability or avidity is lower; as such, we also incubated thymic slices in the presence of 0.1 nM peptide. After 24 h of incubation, we examined the relative ratio of OT‐I thymocytes as compared with overlaid control CD45.1^+^ cells by flow cytometry (Figure 4a, b). In the presence of OVA peptide, we observed a significant decrease in total OT‐I thymocytes on ICOS^+/+^ slices as compared with no peptide control slices, indicating that the OT‐I thymocytes are efficiently deleted in the presence of OVA peptide (Figure 4b, c). However, the extent of negative selection of OT‐I thymocytes is significantly reduced in the absence of ICOSL (Figure 4b, c). In this model, OVA peptide acts like ubiquitous self‐antigen, and TCRs reactive to the peptide can be deleted in both the cortex and the medulla. ICOS expression in OT‐I thymocytes is also elevated during the postselection DP stage and expressed at higher levels on CD8^+^ SP thymocytes (Supplementary figure 4d, e), similar to the expression pattern observed on the polyclonal populations from WT mice (Figure 2a, b). We analyzed whether DP or CD8^+^ SP thymocytes are more resistant to deletion in the absence of ICOSL. We observed that the difference in negative selection efficiency in the presence or absence of ICOSL is slightly less striking in the OT‐I TCR transgenic DP thymocytes (Figure 4d) compared with CD8^+^ SP thymocytes (Figure 4e), and that ICOS–ICOSL interactions may be more important for negative selection when cognate antigen is limited. Therefore, as ICOSL is expressed at a higher level in the thymic medulla, its expression may be more critical for the negative selection of SP thymocytes expressing higher levels of ICOS.

## DISCUSSION

In this study, we suggest that the ICOS–ICOSL pathway may play a role in tuning thymic negative selection. We confirmed that ICOSL is mainly expressed by mTEC^hi^, SIRPα^+^ DCs and thymic B cells, while ICOS is expressed on postselection DP, SP thymocytes and thymic Tregs. Throughout thymic development, ICOS expression generally correlates with the strength of TCR interactions with self‐antigens. In the absence of ICOSL, *in situ* negative selection of MHC‐I‐restricted T cells using a thymic slice model is less efficient.

We present data that suggest that ICOS–ICOSL interactions may play a role in modulating negative selection of CD8‐lineage T cells; whether the same is true for CD4^+^ T cells is not yet clear. We observed a decrease in the percentage of potentially autoreactive PD‐1‐expressing MHC‐I‐restricted CD8^+^ SP thymocytes in the absence of ICOS signaling. In addition, we detected a decrease in deletion of MHC‐I‐restricted antigen‐specific thymocytes in an *in situ* model of negative selection, suggesting that ICOS–ICOSL interactions may play a role in modulating negative selection of CD8‐lineage T cells. By contrast, no consistent differences in mature CD4^+^ SP and thymic Treg populations were noted in the absence of ICOS signaling. Yet, it is possible that ICOS–ICOSL interactions also have an impact on the negative selection of the MHC‐II‐restricted TCR repertoire. MHC‐II‐restricted TCR transgenic models have lower self‐reactivity than that of the polyclonal T‐cell population.^61^ Based on our data, this would also suggest that they may express lower levels of ICOS. Therefore, these TCR transgenic models might be less sensitive to ICOS–ICOSL interactions during thymic selection as compared with their MHC‐I‐restricted TCR transgenic counterparts and additional means are necessary to assess the impact of ICOS signaling on negative selection in the CD4^+^ T‐cell lineage.

In the *in situ* thymic slice system, not only did we observe that negative selection of total and CD8^+^ OT‐I was less efficient in the absence of ICOSL, but we also made similar observations with negative selection of MHC‐I‐restricted DP thymocytes. This is not entirely surprising considering that our results show that postselection DP thymocytes already express ICOS. A small number of thymic DCs in the cortex is sufficient to mediate negative selection,^7^ though SIRPα^+^ DCs with the highest levels of ICOSL are preferentially found at the corticomedullary junction. It is possible that some of the postselection DP thymocytes in our system have already migrated to the medulla as it was previously suggested that DP thymocytes bearing MHC‐I‐restricted TCRs, as in the case of our study, migrate to the medulla prior to becoming CD8^+^ SP thymocytes.^62^ Nevertheless, the differences in negative selection efficiency between WT and ICOSL^−/−^ thymic slices were more evident in the mature CD8^+^ SP thymocyte compartment. Higher expression of ICOS on the SP thymocytes than on the DP thymocytes and more thymic APCs expressing ICOSL could indicate that the expression of ICOSL plays a larger role in the negative selection of thymocytes in the medulla.

We observed that negative selection is less efficient in the absence of ICOSL *in situ*, but it did not completely abrogate deletion of the autoreactive cells. In addition, there are no gross defects in T‐cell development in ICOS‐ and ICOSL‐deficient mice.^55^, ^63^ Although rare, a study reported autoimmune symptoms such as autoimmune arthritis in an ICOS‐deficient patient.^56^ This raises the question as to whether the thymic selection defect we observed in mice may contribute to some autoimmune diseases. However, multiple mouse studies suggest autoimmune symptoms observed in ICOS‐mutant mouse models are largely attributable to weakened Treg functions in the periphery as opposed to increased autoreactive effector T cells. In NOD mice, germline ICOS or ICOSL deficiency dramatically reduces the incidence of type I diabetes, emphasizing a positive costimulatory role of ICOS in pathogenic effector cells.^64^, ^65^ By contrast, in non‐obese diabetic (NOD) mice in which most T cells recognize an autoantigen (BDC2.5 TCR transgenic model), the dominant role of ICOS seems to be the maintenance of Treg cells in the inflamed pancreas to prevent the onset of diabetes.^65^, ^66^, ^67^ Therefore, germline deficiency of ICOS or ICOSL in BDC2.5‐NOD mice accelerates type I diabetes.^65^ Collectively, these data support the notion that the role of ICOS in thymic selection is to fine‐tune the TCR repertoire but the absence of ICOS or ICOSL can be largely compensated by other mechanisms in this context. Indeed, it is possible that multiple cosignaling molecules work together to mediate negative selection; cosignaling through molecules such as CD28, CD40, CD43 and others have been implicated in the deletion of autoreactive thymocytes.^22^, ^32^, ^33^, ^34^, ^68^ Our observations add an additional layer of complexity to the network of cosignaling molecules that may be involved in sculpting the TCR repertoire.

## METHODS

### Mice

C56BL/6, B6.SJL‐Ptprc^a^Pepc^b^/BoyJ (B6SJL), B6.129P2‐Icos^tm1Mak^/J (ICOS^−/−^)^69^ and B6.129P2‐Icosl^tm1Mak^/J (ICOSL^−/−^)^70^ mice were purchased from the Jackson Laboratory (Bar Harbor, ME, USA). C57BL/6‐Tg(OT‐I)‐Rag1<tm1Mom> (OT‐I)^71^, ^72^ mice were obtained through the National Institute of Allergy and Infectious Diseases Exchange Program, National Institutes of Health (Bethesda, MD, USA). SMARTA TCR transgenic^73^ mice were kindly provided by Dr Judith Mandl (McGill University, Montreal, QC, Canada). All mice were bred and maintained in specific pathogen‐free animal facilities at the Maisonneuve‐Rosemont Hospital Research Centre and Institut de Recherches Cliniques de Montréal. Both male and female mice 6–12 weeks of age were used. All animal protocols have been approved by Animal Care Committees at the Maisonneuve‐Rosemont Hospital Research Centre and Institut de Recherches Cliniques de Montréal. Experiments were performed in accordance with the Canadian Council on Animal Care guidelines.

### Cell Isolation

Single‐cell suspensions of thymus were prepared with a tissue grinder. Red blood cells were lysed with Ack lysis buffer (0.15 M NH_4_Cl, 10 mM KHCO_3_, 0.1 mM Na_2_EDTA). For isolation of thymic stromal cells, freshly harvested thymus was cleaned of surrounding connective tissues and cut into small pieces. Wide‐bore 1000 µL tips were used to pipette the thymic pieces up and down until thymocyte release was no longer detectable. The remaining tissue was incubated with Roswell Park Memorial Institute 1640 (RPMI 1640) media (Wisent, St‐Bruno, QC, Canada) supplemented with 10% fetal bovine serum (GE Life Sciences, Pittsburgh, PA, USA), 10 mM HEPES [4‐(2‐hydroxyethyl)‐1‐piperazineethanesulfonic acid; Wisent], 0.32 U mL^−1^ Liberase/thermolysin (Roche, Basel, Switzerland) and 1 mg mL^−1^ DNase I (Thermo Fisher, Waltham, MA, USA) for 30 min at 37°C. Alternatively, the tissue was digested with 0.25 mg mL^−1^ collagenase D (Sigma Aldrich Canada, Oakville, ON, Canada) and 0.1 mg mL^−1^ DNase I (Thermo Fisher). The digestion was stopped with cold phosphate‐buffered saline containing 2% fetal bovine serum. The sample was centrifuged at 200 x *g* for 7 min and the pellet resuspended. All samples were filtered and counted with a hemocytometer using trypan blue; an equal number of cells from each sample was stained for flow cytometry.

### Thymic slices

Thymic selection on thymic slices was performed as previously described.^58^, ^74^, ^75^ Briefly, the thymus of ICOSL^−/−^ and ICOSL^+/+^ controls were harvested, cleaned, embedded in 4% NuSieve GTG agarose (Lonza, Basel, Switzerland) and cut into 500‐µm slices using a vibratome (VT1000S, Leica Biosystems, Wetzlar, Germany). Thymic slices were placed atop 0.4‐μm cell culture inserts (BD Falcon, Corning Inc., Corning, NY, USA) over 1.5 mL of complete Roswell Park Memorial Institute 1640 (RPMI 1640) media containing 2 mM l‐glutamine, 100 IU penicillin and 100 µg mL^−1^ streptomycin (Wisent) and 10% fetal bovine serum (GE Life Sciences) in a 6‐well plate. Total thymocytes from OT‐I mice were stained with CellTrace Violet (Thermo Fisher) according to the manufacturer’s protocol. CellTrace Violet‐stained OT‐I thymocytes were mixed 1:1 with B6SJL thymocytes; 1–3 million thymocytes were overlaid on top of each thymic slice and incubated for 2–3 h at 37°C prior to washing off excess cells that had not migrated into the tissue. For experiments using OVA (SIINFEKL) peptide (AnaSpec, Fremont, CA, USA), OVA peptide was added to the media at the indicated concentrations after the initial 2–3‐h incubation. The slices were further incubated at 37°C for 24 h, at which point the thymic slices were harvested, dissociated with a tissue grinder and analyzed by flow cytometry.

### Antibodies and flow cytometry

Fluorescently labeled anti‐mouse CD4 (GK1.5 and RM4‐5), CD5 (53‐7.3), CD8α (53‐6.7), CD11c (N418), CD25 (PC61), CD45.1 (A20), CD45.2 (104), CD69 (H1.2F3), CD80 (16‐10A1), EpCAM (CD326, G8.8), I‐A/I‐E (M5/114.15.2), ICOS (CD278, C398.4A), ICOSL (CD275, HK5.3), Ly‐51 (6C3), TCRβ (H57‐597), TCR Vα2 (B20.1), SIRPα (CD172a, P84), B220 (RA3‐6B2), H‐2Kb (AF6‐88.5), CD44 (IM7), PD‐1 (CD279, 29F.1A12), CD62L (MEL‐14), CD19 (6D5), CD73 (RTY/11.8), CD11b (M1/70) and Zombie fixable viability dye were purchased from BioLegend (San Diego, CA, USA). Biotinylated anti‐UEA‐1 was purchased from Vector Laboratories (Burlingame, CA, USA), and anti‐Foxp3 (150D/E4) was purchased from eBioscience (Thermo Fisher). Cells were incubated with viability dye according to the manufacturer’s protocol followed by incubation with cell surface antibodies for 20 min at 4°C (10 min in the case of thymic stromal cells). Intranuclear staining of Foxp3 was performed using a Foxp3 staining kit for fixation and permeabilization (eBioscience/Thermo Fisher) according to the manufacturer’s protocol. All data were acquired on an LSR Fortessa X‐20 or LSR II flow cytometer using FACSDiva software and analyzed with FlowJo version 10 (BD Biosciences, San Jose, CA, USA).

### Statistical analysis

Statistical analyses were performed using Prism version 8 (GraphPad, San Diego, CA, USA). Statistical significance is indicated by *P*‐values: *P < 0.05, **P < 0.01, ***P < 0.001.

## CONFLICT OF INTEREST

All authors declare not conflicts of interest.

## AUTHOR CONTRIBUTIONS

**Mengqi Dong:** Conceptualization; Data curation; Formal analysis; Methodology; Writing – original draft; Writing – review & editing. **Jinsam Chang:** Conceptualization; Data curation; Formal analysis; Investigation; Writing – review & editing. **Marie‐Ève Lebel:** Conceptualization; Data curation; Formal analysis; Investigation; Methodology; Supervision; Writing – review & editing. **Noémie Gervais:** Data curation; Methodology. **Marilaine Fournier:** Data curation; Visualization; Writing – review & editing. **Ève Mallet Gauthier:** Methodology; Writing – review & editing. **Woong‐Kyung Suh:** Conceptualization; Funding acquisition; Supervision; Writing – original draft; Writing – review & editing. **Heather J Melichar:** Conceptualization; Funding acquisition; Writing – original draft; Writing – review & editing.

## Data Availability

The data that support the findings of this study are available from the corresponding author upon reasonable request.

## References

[1] Xing Y , Hogquist KA . T‐cell tolerance: central and peripheral. Cold Spring Harb Perspect Biol 2012; 4: a006957. 22661634 10.1101/cshperspect.a006957 PMC3367546

[2] Klein L , Kyewski B , Allen PM , Hogquist KA . Positive and negative selection of the T cell repertoire: what thymocytes see (and don't see). Nat Rev Immunol 2014; 14: 377–391. 24830344 10.1038/nri3667 PMC4757912

[3] Hogquist KA , Jameson SC . The self‐obsession of T cells: how TCR signaling thresholds affect fate 'decisions' and effector function. Nat Immunol 2014; 15: 815–823. 25137456 10.1038/ni.2938 PMC4348363

[4] Klein L , Robey EA , Hsieh CS . Central CD4^+^ T cell tolerance: deletion versus regulatory T cell differentiation. Nat Rev Immunol 2019; 19: 7–18. 30420705 10.1038/s41577-018-0083-6

[5] Stritesky GL , Xing Y , Erickson JR , *et al*. Murine thymic selection quantified using a unique method to capture deleted T cells. Proc Natl Acad Sci USA 2013; 110: 4679–4684. 23487759 10.1073/pnas.1217532110 PMC3606987

[6] Daley SR , Hu DY , Goodnow CC . Helios marks strongly autoreactive CD4^+^ T cells in two major waves of thymic deletion distinguished by induction of PD‐1 or NF‐κB. J Exp Med 2013; 210: 269–285. 23337809 10.1084/jem.20121458 PMC3570102

[7] McCaughtry TM , Baldwin TA , Wilken MS , Hogquist KA . Clonal deletion of thymocytes can occur in the cortex with no involvement of the medulla. J Exp Med 2008; 205: 2575–2584. 18936237 10.1084/jem.20080866 PMC2571932

[8] Sinclair C , Bains I , Yates AJ , Seddon B . Asymmetric thymocyte death underlies the CD4:CD8 T‐cell ratio in the adaptive immune system. Proc Natl Acad Sci USA 2013; 110: E2905–E2914. 23858460 10.1073/pnas.1304859110 PMC3732981

[9] Kishimoto H , Sprent J . Negative selection in the thymus includes semimature T cells. J Exp Med 1997; 185: 263–271. 9016875 10.1084/jem.185.2.263 PMC2196120

[10] Ladi E , Schwickert TA , Chtanova T , *et al*. Thymocyte‐dendritic cell interactions near sources of CCR7 ligands in the thymic cortex. J Immunol 2008; 181: 7014–7023. 18981121 10.4049/jimmunol.181.10.7014

[11] Breed ER , Lee ST , Hogquist KA . Directing T cell fate: how thymic antigen presenting cells coordinate thymocyte selection. Semin Cell Dev Biol 2018; 84: 2–10. 28800929 10.1016/j.semcdb.2017.07.045 PMC5807247

[12] Lebel ME , Coutelier M , Galipeau M , *et al*. Differential expression of tissue‐restricted antigens among mTEC is associated with distinct autoreactive T cell fates. Nat Commun 2020; 11: 3734. 32709894 10.1038/s41467-020-17544-3 PMC7381629

[13] Brennecke P , Reyes A , Pinto S , *et al*. Single‐cell transcriptome analysis reveals coordinated ectopic gene‐expression patterns in medullary thymic epithelial cells. Nat Immunol 2015; 16: 933–941. 26237553 10.1038/ni.3246 PMC4675844

[14] Meredith M , Zemmour D , Mathis D , Benoist C . Aire controls gene expression in the thymic epithelium with ordered stochasticity. Nat Immunol 2015; 16: 942–949. 26237550 10.1038/ni.3247 PMC4632529

[15] Sansom SN , Shikama‐Dorn N , Zhanybekova S , *et al*. Population and single‐cell genomics reveal the Aire dependency, relief from Polycomb silencing, and distribution of self‐antigen expression in thymic epithelia. Genome Res 2014; 24: 1918–1931. 25224068 10.1101/gr.171645.113 PMC4248310

[16] St‐Pierre C , Brochu S , Vanegas JR , *et al*. Transcriptome sequencing of neonatal thymic epithelial cells. Sci Rep 2013; 3: 1860. 23681267 10.1038/srep01860 PMC3656389

[17] Koble C , Kyewski B . The thymic medulla: a unique microenvironment for intercellular self‐antigen transfer. J Exp Med 2009; 206: 1505–1513. 19564355 10.1084/jem.20082449 PMC2715082

[18] Gallegos AM , Bevan MJ . Central tolerance to tissue‐specific antigens mediated by direct and indirect antigen presentation. J Exp Med 2004; 200: 1039–1049. 15492126 10.1084/jem.20041457 PMC2211843

[19] Li J , Park J , Foss D , Goldschneider I . Thymus‐homing peripheral dendritic cells constitute two of the three major subsets of dendritic cells in the steady‐state thymus. J Exp Med 2009; 206: 607–622. 19273629 10.1084/jem.20082232 PMC2699131

[20] Hadeiba H , Lahl K , Edalati A , *et al*. Plasmacytoid dendritic cells transport peripheral antigens to the thymus to promote central tolerance. Immunity 2012; 36: 438–450. 22444632 10.1016/j.immuni.2012.01.017 PMC3315699

[21] Bonasio R , Scimone ML , Schaerli P , *et al*. Clonal deletion of thymocytes by circulating dendritic cells homing to the thymus. Nat Immunol 2006; 7: 1092–1100. 16951687 10.1038/ni1385

[22] Yamano T , Nedjic J , Hinterberger M , *et al*. Thymic B cells are licensed to present self antigens for central T cell tolerance induction. Immunity 2015; 42: 1048–1061. 26070482 10.1016/j.immuni.2015.05.013

[23] Perera J , Meng L , Meng F , Huang H . Autoreactive thymic B cells are efficient antigen‐presenting cells of cognate self‐antigens for T cell negative selection. Proc Natl Acad Sci USA 2013; 110: 17011–17016. 24082098 10.1073/pnas.1313001110 PMC3801014

[24] Yamano T , Steinert M , Klein L . Thymic B cells and central T cell tolerance. Front Immunol 2015; 6: 376. 26257742 10.3389/fimmu.2015.00376 PMC4510420

[25] Page DM , Kane LP , Allison JP , Hedrick SM . Two signals are required for negative selection of CD4^+^CD8^+^ thymocytes. J Immunol 1993; 151: 1868–1880. 7688388

[26] Noel PJ , Alegre ML , Reiner SL , Thompson CB . Impaired negative selection in CD28‐deficient mice. Cell Immunol 1998; 187: 131–138. 9732701 10.1006/cimm.1998.1332

[27] Pobezinsky LA , Angelov GS , Tai X , *et al*. Clonal deletion and the fate of autoreactive thymocytes that survive negative selection. Nat Immunol 2012; 13: 569–578. 22544394 10.1038/ni.2292 PMC3362677

[28] Punt JA , Osborne BA , Takahama Y , Sharrow SO , Singer A . Negative selection of CD4^+^CD8^+^ thymocytes by T cell receptor‐induced apoptosis requires a costimulatory signal that can be provided by CD28. J Exp Med 1994; 179: 709–713. 8294878 10.1084/jem.179.2.709 PMC2191361

[29] Jones LA , Izon DJ , Nieland JD , Linsley PS , Kruisbeek AM . CD28‐B7 interactions are not required for intrathymic clonal deletion. Int Immunol 1993; 5: 503–512. 7686392 10.1093/intimm/5.5.503

[30] Shahinian A , Pfeffer K , Lee KP , *et al*. Differential T cell costimulatory requirements in CD28‐deficient mice. Science 1993; 261: 609–612. 7688139 10.1126/science.7688139

[31] Walunas TL , Sperling AI , Khattri R , Thompson CB , Bluestone JA . CD28 expression is not essential for positive and negative selection of thymocytes or peripheral T cell tolerance. J Immunol 1996; 156: 1006–1013. 8557973

[32] Kishimoto H , Sprent J . Several different cell surface molecules control negative selection of medullary thymocytes. J Exp Med 1999; 190: 65–73. 10429671 10.1084/jem.190.1.65 PMC2195556

[33] Li R , Page DM . Requirement for a complex array of costimulators in the negative selection of autoreactive thymocytes *in vivo* . J Immunol 2001; 166: 6050–6056. 11342622 10.4049/jimmunol.166.10.6050

[34] Page DM . Cutting edge: thymic selection and autoreactivity are regulated by multiple coreceptors involved in T cell activation. J Immunol 1999; 163: 3577–3581. 10490949

[35] Amakawa R , Hakem A , Kundig TM , *et al*. Impaired negative selection of T cells in Hodgkin's disease antigen CD30‐deficient mice. Cell 1996; 84: 551–562. 8598042 10.1016/s0092-8674(00)81031-4

[36] Page DM , Roberts EM , Peschon JJ , Hedrick SM . TNF receptor‐deficient mice reveal striking differences between several models of thymocyte negative selection. J Immunol 1998; 160: 120–133. 9551964

[37] Wikenheiser DJ , Stumhofer JS . ICOS co‐stimulation: friend or foe? Front Immunol 2016; 7: 304. 27559335 10.3389/fimmu.2016.00304 PMC4979228

[38] Panneton V , Chang J , Witalis M , Li J , Suh WK . Inducible T‐cell co‐stimulator: signaling mechanisms in T follicular helper cells and beyond. Immunol Rev 2019; 291: 91–103. 31402504 10.1111/imr.12771

[39] Buus TB , Schmidt JD , Bonefeld CM , Geisler C , Lauritsen JPH . Development of interleukin‐17‐producing Vγ2^+^γδ T cells is reduced by ICOS signaling in the thymus. Oncotarget 2016; 7: 19341–19354. 27235509 10.18632/oncotarget.8464 PMC4991387

[40] Chung Y , Nurieva R , Esashi E , *et al*. A critical role of costimulation during intrathymic development of invariant NK T cells. J Immunol 2008; 180: 2276–2283. 18250436 10.4049/jimmunol.180.4.2276

[41] Nazzal D , Gradolatto A , Truffault F , Bismuth J , Berrih‐Aknin S . Human thymus medullary epithelial cells promote regulatory T‐cell generation by stimulating interleukin‐2 production via ICOS ligand. Cell Death Dis 2014; 5: e1420. 25210803 10.1038/cddis.2014.377 PMC4540205

[42] Hemmers S , Schizas M , Azizi E , *et al*. IL‐2 production by self‐reactive CD4 thymocytes scales regulatory T cell generation in the thymus. J Exp Med 2019; 216: 2466–2478. 31434685 10.1084/jem.20190993 PMC6829602

[43] Landuyt AE , Klocke BJ , Colvin TB , Schoeb TR , Maynard CL . Cutting edge: ICOS‐deficient regulatory T cells display normal induction of *Il10* but readily downregulate expression of Foxp3. J Immunol 2019; 202: 1039–1044. 30642977 10.4049/jimmunol.1801266 PMC6363853

[44] White AJ , Jenkinson WE , Cowan JE , *et al*. An essential role for medullary thymic epithelial cells during the intrathymic development of invariant NKT cells. J Immunol 2014; 192: 2659–2666. 24510964 10.4049/jimmunol.1303057 PMC3948113

[45] Autengruber A , Gereke M , Hansen G , Hennig C , Bruder D . Impact of enzymatic tissue disintegration on the level of surface molecule expression and immune cell function. Eur J Microbiol Immunol (Bp) 2012; 2: 112–120. 24672679 10.1556/EuJMI.2.2012.2.3 PMC3956959

[46] Miragaia RJ , Zhang X , Gomes T , *et al*. Single‐cell RNA‐sequencing resolves self‐antigen expression during mTEC development. Sci Rep 2018; 8: 685. 29330484 10.1038/s41598-017-19100-4 PMC5766627

[47] Heng TS , Painter MW , Immunological Genome Project C . The Immunological Genome Project: networks of gene expression in immune cells. Nat Immunol 2008; 9: 1091–1094. 18800157 10.1038/ni1008-1091

[48] Liang L , Porter EM , Sha WC . Constitutive expression of the B7h ligand for inducible costimulator on naive B cells is extinguished after activation by distinct B cell receptor and interleukin 4 receptor‐mediated pathways and can be rescued by CD40 signaling. J Exp Med 2002; 196: 97–108. 12093874 10.1084/jem.20020298 PMC2194020

[49] Xing Y , Wang X , Jameson SC , Hogquist KA . Late stages of T cell maturation in the thymus involve NF‐κB and tonic type I interferon signaling. Nat Immunol 2016; 17: 565–573. 27043411 10.1038/ni.3419 PMC4837029

[50] Thiault N , Darrigues J , Adoue V , *et al*. Peripheral regulatory T lymphocytes recirculating to the thymus suppress the development of their precursors. Nat Immunol 2015; 16: 628–634. 25939024 10.1038/ni.3150

[51] Owen DL , Mahmud SA , Sjaastad LE , *et al*. Thymic regulatory T cells arise via two distinct developmental programs. Nat Immunol 2019; 20: 195–205. 30643267 10.1038/s41590-018-0289-6 PMC6650268

[52] Azzam HS , Grinberg A , Lui K , *et al*. CD5 expression is developmentally regulated by T cell receptor (TCR) signals and TCR avidity. J Exp Med 1998; 188: 2301–2311. 9858516 10.1084/jem.188.12.2301 PMC2212429

[53] Bellinghausen I , Klostermann B , Bottcher I , Knop J , Saloga J . Importance of the inducible costimulator molecule for the induction of allergic immune responses and its decreased expression on T helper cells after venom immunotherapy. Immunology 2004; 112: 80–86. 15096187 10.1111/j.1365-2567.2004.01845.x PMC1782455

[54] McAdam AJ , Chang TT , Lumelsky AE , *et al*. Mouse inducible costimulatory molecule (ICOS) expression is enhanced by CD28 costimulation and regulates differentiation of CD4^+^ T cells. J Immunol 2000; 165: 5035–5040. 11046032 10.4049/jimmunol.165.9.5035

[55] Dong C , Juedes AE , Temann UA , *et al*. ICOS co‐stimulatory receptor is essential for T‐cell activation and function. Nature 2001; 409: 97–101. 11343121 10.1038/35051100

[56] Takahashi N , Matsumoto K , Saito H , *et al*. Impaired CD4 and CD8 effector function and decreased memory T cell populations in ICOS‐deficient patients. J Immunol 2009; 182: 5515–5527. 19380800 10.4049/jimmunol.0803256

[57] Dzhagalov IL , Chen KG , Herzmark P , Robey EA . Elimination of self‐reactive T cells in the thymus: a timeline for negative selection. PLoS Biol 2013; 11: e1001566. 23700386 10.1371/journal.pbio.1001566 PMC3660248

[58] Zhou TA , Hsu CL , Dzhagalov IL . Testing the efficiency and kinetics of negative selection using thymic slices. Methods Mol Biol 2020; 2111: 205–219. 31933210 10.1007/978-1-0716-0266-9_17

[59] Au‐Yeung BB , Melichar HJ , Ross JO , *et al*. Quantitative and temporal requirements revealed for Zap70 catalytic activity during T cell development. Nat Immunol 2014; 15: 687–694. 24908390 10.1038/ni.2918 PMC4095875

[60] Melichar HJ , Ross JO , Herzmark P , Hogquist KA , Robey EA . Distinct temporal patterns of T cell receptor signaling during positive versus negative selection in situ. Sci Signal 2013; 6: ra92. 24129702 10.1126/scisignal.2004400 PMC4078262

[61] Mandl JN , Monteiro JP , Vrisekoop N , Germain RN . T cell‐positive selection uses self‐ligand binding strength to optimize repertoire recognition of foreign antigens. Immunity 2013; 38: 263–274. 23290521 10.1016/j.immuni.2012.09.011 PMC3785078

[62] Ross JO , Melichar HJ , Au‐Yeung BB , *et al*. Distinct phases in the positive selection of CD8^+^ T cells distinguished by intrathymic migration and T‐cell receptor signaling patterns. Proc Natl Acad Sci USA 2014; 111: E2550–E2558. 24927565 10.1073/pnas.1408482111 PMC4078834

[63] Burmeister Y , Lischke T , Dahler AC , *et al*. ICOS controls the pool size of effector‐memory and regulatory T cells. J Immunol 2008; 180: 774–782. 18178815 10.4049/jimmunol.180.2.774

[64] Hawiger D , Tran E , Du W , *et al*. ICOS mediates the development of insulin‐dependent diabetes mellitus in nonobese diabetic mice. J Immunol 2008; 180: 3140–3147. 18292537 10.4049/jimmunol.180.5.3140

[65] Prevot N , Briet C , Lassmann H , *et al*. Abrogation of ICOS/ICOS ligand costimulation in NOD mice results in autoimmune deviation toward the neuromuscular system. Eur J Immunol 2010; 40: 2267–2276. 20544729 10.1002/eji.201040416

[66] Herman AE , Freeman GJ , Mathis D , Benoist C . CD4^+^CD25^+^ T regulatory cells dependent on ICOS promote regulation of effector cells in the prediabetic lesion. J Exp Med 2004; 199: 1479–1489. 15184501 10.1084/jem.20040179 PMC2211778

[67] Kornete M , Sgouroudis E , Piccirillo CA . ICOS‐dependent homeostasis and function of Foxp3^+^ regulatory T cells in islets of nonobese diabetic mice. J Immunol 2012; 188: 1064–1074. 22227569 10.4049/jimmunol.1101303

[68] Williams JA , Zhang J , Jeon H , *et al*. Thymic medullary epithelium and thymocyte self‐tolerance require cooperation between CD28‐CD80/86 and CD40‐CD40L costimulatory pathways. J Immunol 2014; 192: 630–640. 24337745 10.4049/jimmunol.1302550 PMC3897934

[69] Tafuri A , Shahinian A , Bladt F , *et al*. ICOS is essential for effective T‐helper‐cell responses. Nature 2001; 409: 105–109. 11343123 10.1038/35051113

[70] Mak TW , Shahinian A , Yoshinaga SK , *et al*. Costimulation through the inducible costimulator ligand is essential for both T helper and B cell functions in T cell‐dependent B cell responses. Nat Immunol 2003; 4: 765–772. 12833154 10.1038/ni947

[71] Mombaerts P , Iacomini J , Johnson RS , *et al*. RAG‐1‐deficient mice have no mature B and T lymphocytes. Cell 1992; 68: 869–877. 1547488 10.1016/0092-8674(92)90030-g

[72] Hogquist KA , Jameson SC , Heath WR , *et al*. T cell receptor antagonist peptides induce positive selection. Cell 1994; 76: 17–27. 8287475 10.1016/0092-8674(94)90169-4

[73] Oxenius A , Bachmann MF , Zinkernagel RM , Hengartner H . Virus‐specific MHC‐class II‐restricted TCR‐transgenic mice: effects on humoral and cellular immune responses after viral infection. Eur J Immunol 1998; 28: 390–400. 9485218 10.1002/(SICI)1521-4141(199801)28:01<390::AID-IMMU390>3.0.CO;2-O

[74] Ross JO , Melichar HJ , Halkias J , Robey EA . Studying T cell development in thymic slices. Methods Mol Biol 2016; 1323: 131–140. 26294404 10.1007/978-1-4939-2809-5_11

[75] Sood A , Dong M , Melichar HJ . Preparation and applications of organotypic thymic slice cultures. J Vis Exp 2016; 114: e54355. 10.3791/54355 PMC5091756 27585240

