# Supplementary material for: The ICOS–ICOSL pathway tunes thymic selection
Source: Immunol Cell Biol. 2022 Jan 23;100(3):205–17. doi: 10.1111/imcb.12520 (PMC9304562; doi:10.1111/imcb.12520)
Supplement: Supplementary file 1 [file IMCB-100-205-s001.pdf]

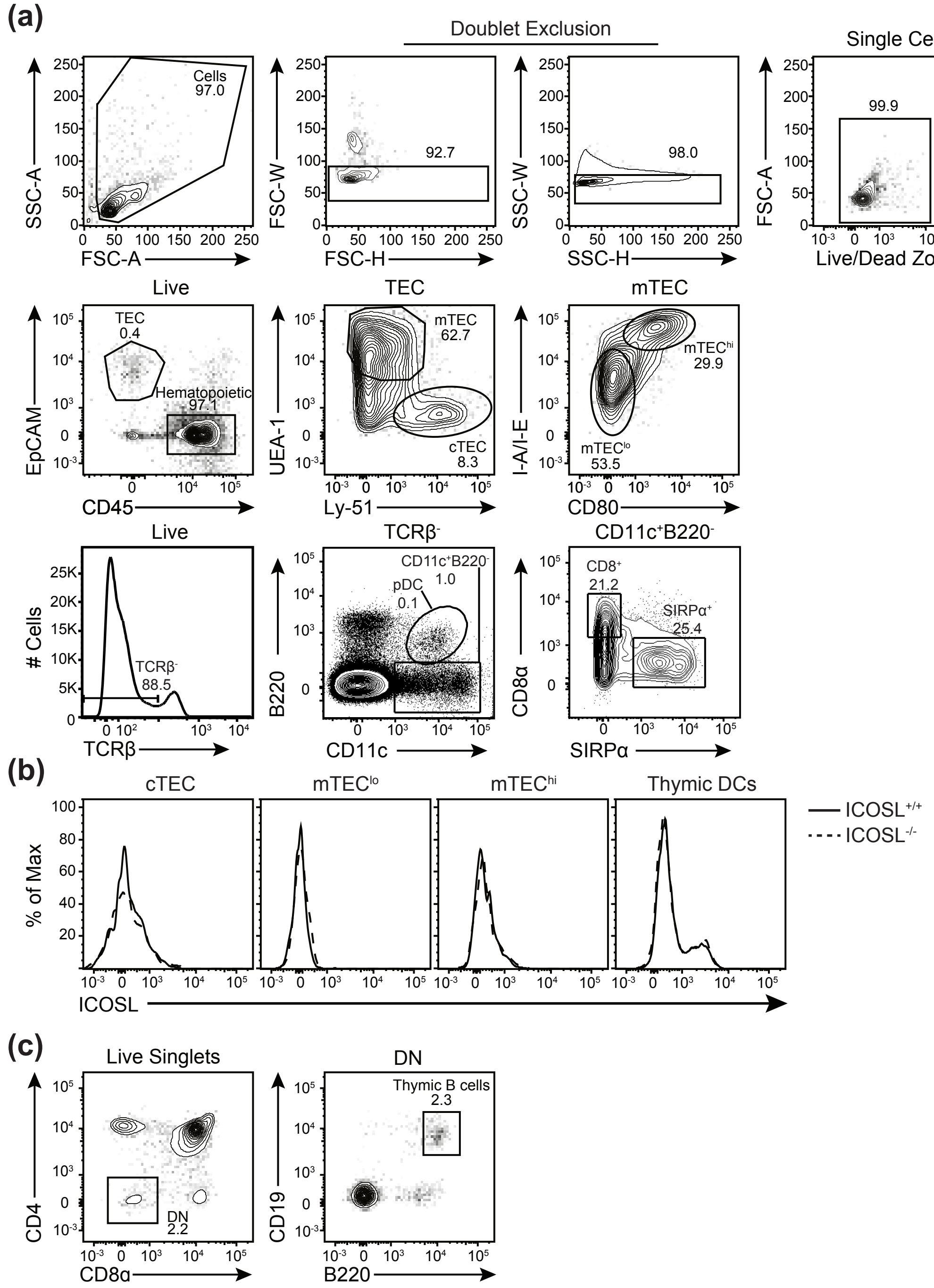

Supplementary figure 1. Gating strategies for ICOSL expression on thymic antigen presenting cells. Representative flow plots depicting gating strategies for thymic epithelial cells (TEC), thymic dendritic cells (DC), and thymic B cells. **(a)** After excluding doublets, TEC were gated on live cells that are EpCAM^{+}CD45^{-}. Medullary TEC (mTEC) are UEA-1^{+}Ly-51^{-}, while cortical TEC (cTEC) are UEA-1^{-}Ly-51^{+}. mTEC are further divided into mTEC^{hi} (CD80^{hi}I-A/I-E^{hi}) and mTEC^{lo} (CD80^{lo}I-A/I-E^{lo}) subsets. Plasmacytoid DC (pDC) were identified as TCRβ^{-}CD11c^{int}B220^{+} while CD8^{+} conventional DC were TCR β^{-}CD11c^{+}CD8^{+}SIRPα^{-} and SIRPα^{+} conventional DC were TCRβ^{-}CD11c^{+}CD8^{-}SIRPα^{+}. **(b)** Using collagenase to digest thymic tissue appears to cleave ICOSL from the surface of cells as there is similar expression of ICOSL on cells from ICOSL$^{+/+}$ and ICOSL$^{-/-}$ mice. **(c)** After excluding doublets, thymic B cells were first gated on double negative (DN, CD4^{-}CD8^{-}) thymocytes, followed by gating for CD19^{+}B220^{+}.

(a)

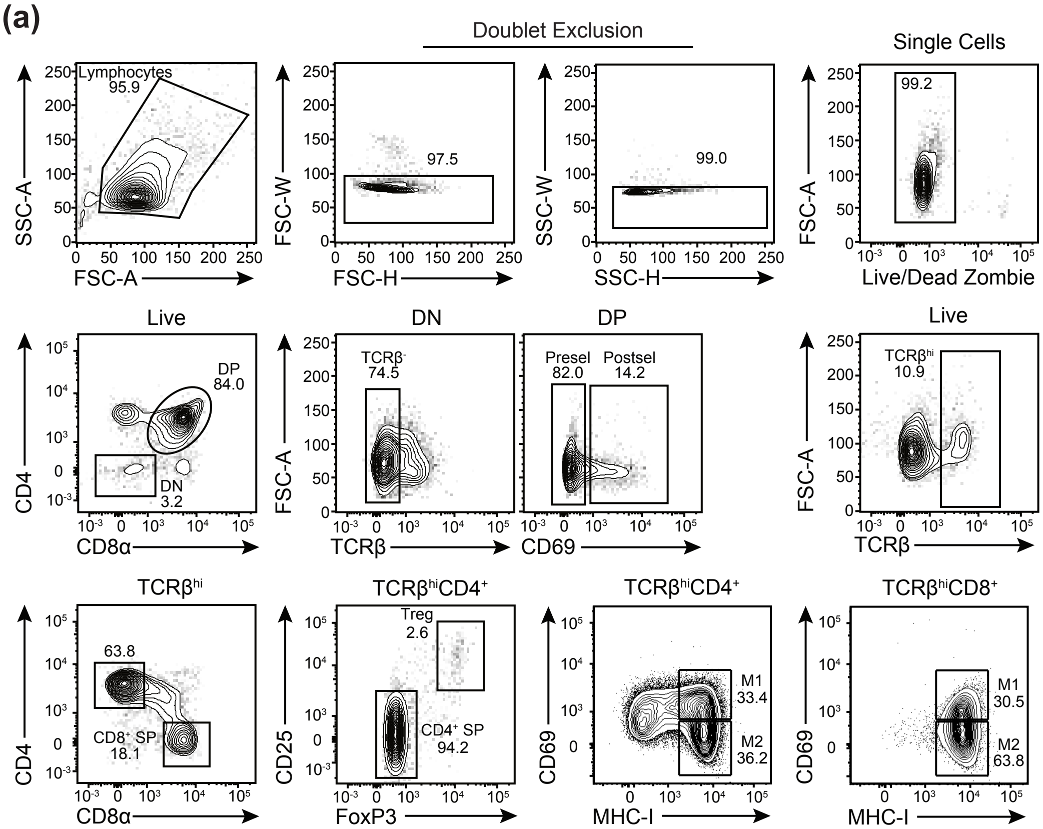

(b)

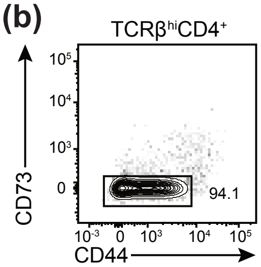

Supplementary figure 2. Gating strategies for analysis of ICOS expression on thymocyte subsets. Representative flow plots depicting gating strategies in the thymus for **(a)** double negative (DN, TCRβ$^-$CD4$^-$CD8$^-$), pre-selection double positive (Presel DP, CD4$^+$CD8$^+$CD69$^-$), post-selection DP (Postsel DP, CD4$^+$CD8$^+$CD69$^+$), CD8$^+$ single positive (SP, TCRβ^{hi}CD8$^+$), and CD4$^+$ SP (TCRβ^{hi}CD4$^+$CD25$^-$FoxP3$^-$) thymocytes, thymic regulatory T cells (Treg, TCRβ ^{hi}CD4$^+$CD25$^+$FoxP3$^+$) as well as the maturation stages M1 (CD69$^+$MHC-I$^+$) and M2 (CD69$^-$MHC-I$^+$) for CD4$^+$ SP and CD8$^+$ SP thymocytes; and **(b)** gating on *de novo* generated thymic CD4$^+$ T cells (including Tregs).

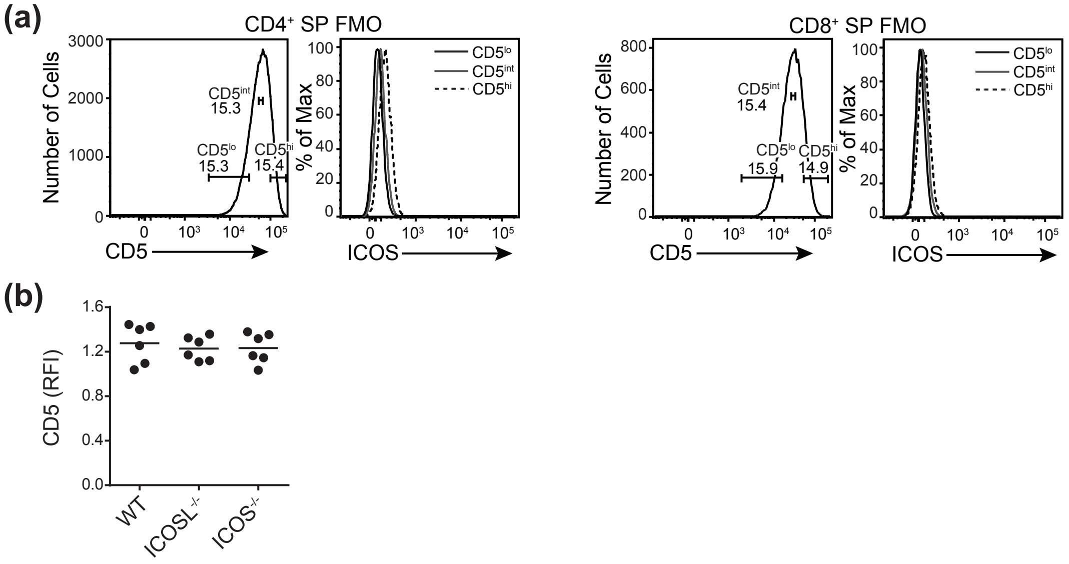

Supplementary figure 3. Influence of T cell receptor signaling strength on ICOS expression on thymocyte subsets. **(a)** Fluorescence minus one (FMO) control for ICOS expression on $CD5^{lo}$, $CD5^{int}$ and $CD5^{hi}$ $CD4^+$ and $CD8^+$ single positive (SP) thymocytes. **(b)** Quantification of CD5 expression on $TCR\beta^{hi}CD4^+CD25^+FoxP3^+$ thymic regulatory T (Treg) cells. The relative fluorescence intensity (RFI) for CD5 is normalized to the median fluorescence intensity of $CD4^+$ SP in each experiment.

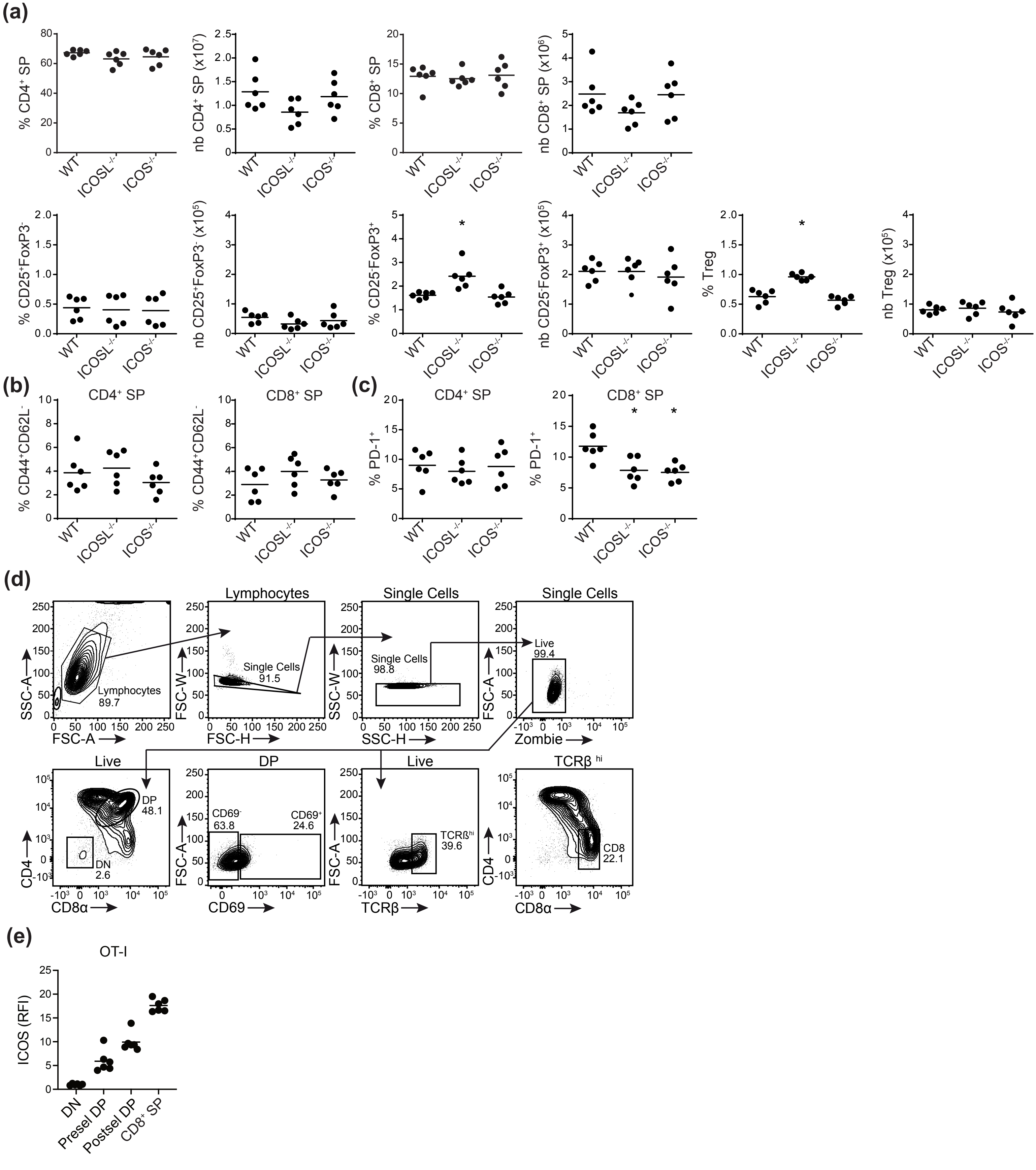

Supplementary figure 4. Potential impact of ICOS signaling on the negative selection of CD8-lineage thymocytes. **(a)** Quantification of the percentages and cell numbers of $CD4^+$ single positive (SP) and $CD8^+$ SP thymocytes, $CD25^+FoxP3^-$ and $CD25^-FoxP3^+$ thymic regulatory T (Treg) cell precursors and $Foxp3^+CD25^+$ Tregs. **(b)** Percentage of $CD44^+CD62L^-$ cells among $CD4^+$ SP and $CD8^+$ SP thymocytes. **(c)** Percentage of PD-1^+ cells among the $CD4^+$ SP and $CD8^+$ SP thymocyte populations. * $P < 0.05$ of ICOSL- or ICOS-deficient mice as compared to wild-type (WT) controls, ANOVA followed by multiple comparisons test **(a)**, **(b)**, and **(c)**. **(d)** Representative flow plots depicting gating strategies for thymic developmental intermediates in OT-I TCR transgenic mice. **(e)** ICOS expression is developmentally regulated on OT-I TCR transgenic thymocytes.
